# *Mycobacterium tuberculosis* Infection Interferes with HIV Vaccination in Mice

**DOI:** 10.1371/journal.pone.0041205

**Published:** 2012-07-23

**Authors:** Lech Ignatowicz, Jolanta Mazurek, Chaniya Leepiyasakulchai, Markus Sköld, Jorma Hinkula, Gunilla Källenius, Andrzej Pawlowski

**Affiliations:** 1 Department of Microbiology, Tumor and Cell Biology, Karolinska Institutet, Stockholm, Sweden; 2 Department of Clinical and Experimental Medicine, Linköping University, Linköping, Sweden; 3 Department of Clinical Science and Education, Karolinska Institutet, Stockholm, Sweden; University of Cape Town, South Africa

## Abstract

Tuberculosis (TB) has emerged as the most prominent bacterial disease found in human immunodeficiency virus (HIV)-positive individuals worldwide. Due to high prevalence of asymptomatic *Mycobacterium tuberculosis* (*Mtb*) infections, the future HIV vaccine in areas highly endemic for TB will often be administrated to individuals with an ongoing *Mtb* infection. The impact of concurrent *Mtb* infection on the immunogenicity of a HIV vaccine candidate, MultiHIV DNA/protein, was investigated in mice. We found that, depending on the vaccination route, mice infected with *Mtb* before the administration of the HIV vaccine showed impairment in both the magnitude and the quality of antibody and T cell responses to the vaccine components p24Gag and gp160Env. Mice infected with *Mtb* prior to intranasal HIV vaccination exhibited reduced p24Gag-specific serum IgG and IgA, and suppressed gp160Env-specific serum IgG as compared to respective titers in uninfected HIV-vaccinated controls. Importantly, in *Mtb*-infected mice that were HIV-vaccinated by the intramuscular route the virus neutralizing activity in serum was significantly decreased, relative to uninfected counterparts. In addition mice concurrently infected with *Mtb* had fewer p24Gag-specific IFN-γ-expressing T cells and multifunctional T cells in their spleens. These results suggest that *Mtb* infection might interfere with the outcome of prospective HIV vaccination in humans.

## Introduction

Despite recent advances in highly active anti-retroviral therapy, human immunodeficiency virus (HIV) infections and the resulting acquired immunodeficiency syndrome (AIDS) remain an important cause of morbidity and mortality worldwide with 2.6 million new cases and 1.8 million deaths reported in 2009 [1]. Therefore, it is widely acknowledged that a safe and effective HIV prophylactic vaccine would be the best long-term measure to bring the HIV/AIDS epidemics under control.

It has been suggested that the effectiveness of vaccines in the population is affected by several factors such as age [2], malnutrition [3], and concurrent infections [4]–[9]. One of the factors that could potentially affect HIV vaccination efficacy is high prevalence of tuberculosis (TB) in HIV endemic regions. Over 90% of the world’s HIV/AIDS cases are in Africa where TB is the leading cause of HIV-related mortality [10]. The HIV and TB epidemics fuel each other [11] and the relationship between HIV and *Mycobacterium tuberculosis* (*Mtb*) infection in co-infected individuals has been shown to be synergistic; latent *Mtb* infection is activated by HIV-induced immunodeficiency and latent HIV in proviral form is triggered by TB-induced immune activation [12], [13]. In addition, TB impairs recovery of immune system in HIV-infected patients undergoing anti-retroviral therapy [14].

Studies of long-term non-progressors, a small subset of HIV-1 infected individuals who have stable CD4 T cell counts for more than 5 years without retroviral therapy [15], firmly suggest that an effective immune response helps control the infection and disease. These studies imply that, by analogy to natural HIV infection in long-term non-progressors, an efficient HIV vaccine should elicit cytotoxic T cell responses [16], and multifunctional T cells that produce multiple cytokines in response to HIV antigens [17]. In addition to cell-mediated immunity, the HIV vaccine should evoke early and robust broadly virus-neutralizing antibodies [18] similar to those identified in a subset of HIV-1 infected subjects [19]. It is also considered important that, in order to prevent the infection or reduce the infectious inoculum, the HIV vaccine should induce immune responses at mucosal surfaces, which represent sites of HIV entry [20], [21].

An extensive search for a HIV vaccine has resulted in a large number of vaccine candidates that in laboratory animals elicited immune responses against HIV antigens [22]. Based on results of immunogenicity and protection studies in non-human primates several promising HIV vaccine candidates were taken to clinical trials. To date, out of several vaccine candidates investigated in clinical phase II/III trials, only one showed moderate level of protective efficacy. Thus, although level of protection afforded by this vaccine was unsatisfactory, the trial demonstrated that construction of an effective HIV vaccine is possible [23]. Nevertheless, future HIV vaccine studies should, in addition to defining protective immune responses, also focus on factors that could interfere with vaccine-induced protection.

One of the overlooked issues that have potential impact on HIV vaccine development stems from the fact that geographical areas with the highest prevalence of HIV and *Mtb* infections overlap. Consequently, future HIV vaccine will often be administered to individuals harboring latent or undiagnosed active TB. Several acute or chronic infections, such as measles, malaria, and helminthes have previously been found to interfere with efficacy of vaccination against unrelated pathogens [4]–[9]. In contrast, the impact of TB on HIV vaccine efficacy has not yet been addressed in preclinical studies, despite the high prevalence of TB in HIV vaccine target populations.

In this study we investigated the effect of concurrent chronic *Mtb* infection on immunogenicity of a HIV DNA/protein vaccine candidate that has generated promising results in a mouse model [24]. We found that both the magnitude and the quality of antibody and T cell responses to such vaccine were impaired by *Mtb* infection.

## Results

### Concurrent *Mtb* Infection Impairs IgA Levels Induced by the HIV Vaccination

The humoral response mediated by IgA at mucosal surfaces may help prevent HIV infection or reduce the viral load [20]. We therefore assessed the impact of ongoing *Mtb* infection on IgA responses to HIV vaccination by examining the relative levels of p24Gag and gp160Env-specific IgA in vaginal secretions and sera from *Mtb*-infected and MultiHIV DNA/protein-vaccinated mice, as compared to uninfected vaccinated animals.

**Figure 1:**
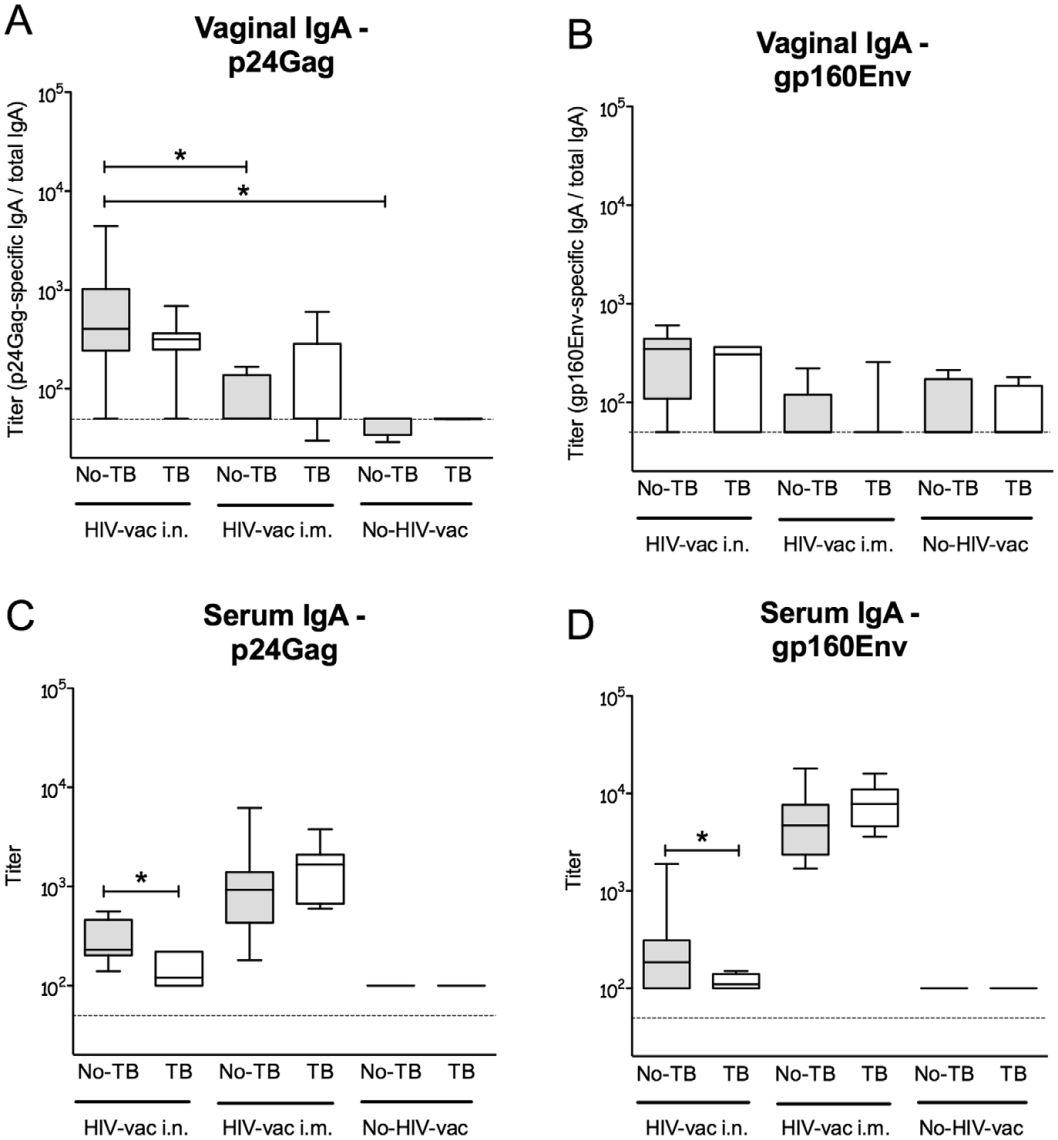
HIV-specific vaginal secretory and serum IgA in uninfected and *Mtb*-infected C57BL/6 mice post-vaccination with MultiHIV DNA/protein. Uninfected C57BL/6 mice, or *Mtb* aerosol-infected mice, were immunized and boosted with MultiHIV DNA/protein as described in Materials and Methods. Vaginal secretions were collected 4 wk post-vaccination and HIV p24Gag-specific and gp160Env-specific IgA were determined in vaginal washings (A, B) and serum (C, D) as described in Materials and Methods. The median endpoint titer of 6–8 mice/group from one individual experiment is shown as a solid line. The box defines the 75th and 25th percentiles and the whiskers define the maximum and minimum values. Dashed line indicates the ELISA sensitivity threshold. (*: P<0.05). HIV-specific IgA levels were examined in two separate experiments.

Importantly, HIV vaccination of mice via the intranasal (i.n.) route resulted in moderately high levels of p24Gag- and gp160Env-specific vaginal IgA 4 weeks (wk) post-vaccination (Fig. 1A and B). Compared to unifected mice, the titers seemed lower in *Mtb*-infected animals, although the difference did not reach statistical significance. As expected, intramuscular (i.m.) vaccination did not induce any detectable IgA in vaginal secretions. The HIV vaccine-induced serum IgA levels were higher following i.m. vaccination when compared to i.n. vaccination (Figure 1C and D). While the anti-p24Gag and anti-gp160Env serum IgA levels elicited by i.m. vaccination were not modified by prior *Mtb* infection, those induced by i.n. vaccination were significantly suppressed in *Mtb*-infected mice (P<0.05).

Thus, *Mtb* infection was shown to diminish HIV-specific IgA responses at mucosal surfaces.

### Concurrent *Mtb* Infection Reduces Serum Antibody Responses to the MultiHIV DNA/Protein Vaccine Administered I.N

To determine whether concurrent *Mtb* infection affects IgG responses to HIV vaccination, we investigated serum p24Gag- and gp160Env-specific IgG in *Mtb*-infected and uninfected mice after MultiHIV DNA/protein immunization.

**Figure 2:**
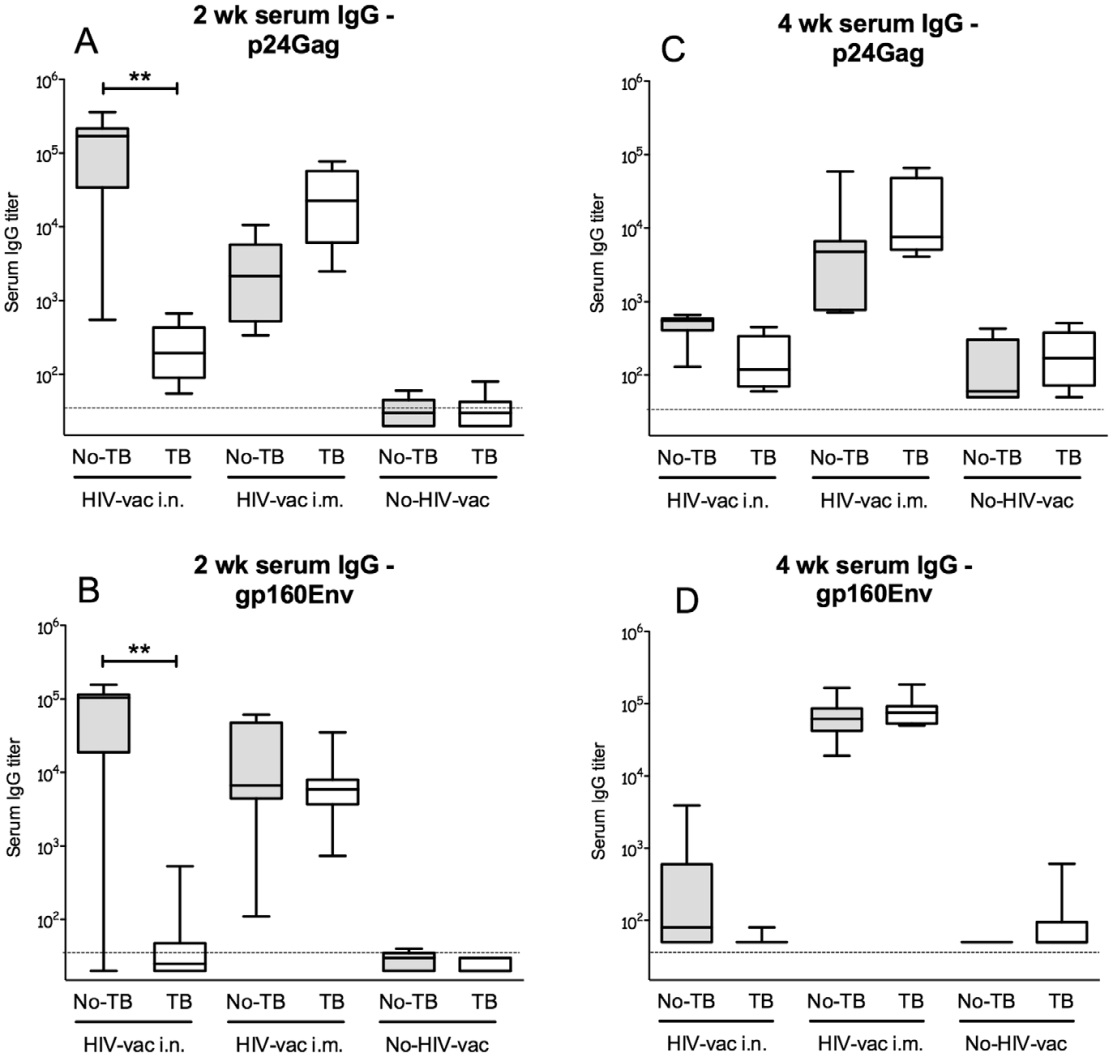
Effect of *Mtb* infection on HIV-specific serum IgG titers induced by MultiHIV DNA vaccination followed by protein boost. Uninfected or low-dose *Mtb* aerosol-infected (7 wk post infection) C57BL/6 mice were vaccinated i.n. or i.m. with MultiHIV DNA encoding HIV-1 subtype B gp160Env, p37Gag, Nef, Tat, and Rev in N3 adjuvant followed by two booster inoculations of recombinant HIV proteins (gp160Env, p24Gag, Tat, and Nef) in L3 adjuvant. HIV-specific serum IgG levels were measured 2 (A, B) and 4 wk (C, D) post-vaccination with HIV antigen-ELISA using p24Gag (A, C) and gp160Env (B, D) as coating antigens, as described in Materials and Methods. Median endpoint titer of 6–8 mice/group from one individual experiment is shown as a solid line. The box defines the 75th and 25th percentiles and the whiskers define the maximum and minimum values. Dashed line indicates the ELISA sensitivity threshold. (*: P<0.05; **: P<0.01). The IgG titers were examined in two separate experiments.

Two wk post-vaccination (after the second protein boost) uninfected mice had high serum levels of anti-p24Gag IgG (Figure 2A) and anti-gp160Env IgG (Figure 2B). The IgG titers in mice vaccinated by the i.n. route were over 1 log higher than those in mice vaccinated by the i.m. route, but the difference did not reach statistical significance. Strikingly, in *Mtb*-infected and i.n. vaccinated mice, anti-p24Gag serum IgG were reduced by almost 3 logs (Figure 2A; P<0.01) and anti-gp160Env serum IgG were virtually absent (Figure 2B; P<0.01), as compared to the serum IgG levels in uninfected mice. Unlike serum IgG responses elicited by i.n. vaccination, those induced by i.m. vaccination were not impaired by the concurrent *Mtb* infection.

Both p24Gag- and gp160Env-specific serum IgG elicited by i.n. vaccination decreased significantly 4 wk post-infection (Figure 2C and D; P<0.05). Despite this reduction the trend for compromised HIV antibody responses in *Mtb*-infected relative to uninfected animals could still be observed. Conversely, at 4 wk, p24Gag- and gp160Env-specific serum IgG levels induced by the i.m. vaccination remained as high as at 2 wk and were not affected by pre-existing *Mtb* infection.

Of note, MultiHIV DNA/protein elicited moderately high anti-Nef and anti-Tat serum IgG that were not altered by a concurrent *Mtb* infection, irrespective of the vaccination route (Figure S1).

Our results show that, in addition to affecting the specific mucosal IgA response, *Mtb* infection can, depending on the vaccination route, significantly reduce the serum IgG titers induced by a HIV vaccine.

### Concurrent *Mtb* Infection Reduces HIV Vaccine-induced Virus Neutralizing Activity in Sera of Mice Vaccinated by the I.M. Route

Studies of long-term non-progressors indicate that more important than the amount of HIV-specific antibodies is their ability to neutralize the virus [15], [19]. In order to assess the impact of prior *Mtb* infection on the quality of antibody responses to subsequent HIV vaccination, we investigated heterologous HIV neutralizing activity in 4 wk post-vaccination sera from uninfected or *Mtb*-infected and MultiHIV DNA/protein-vaccinated mice.

**Figure 3:**
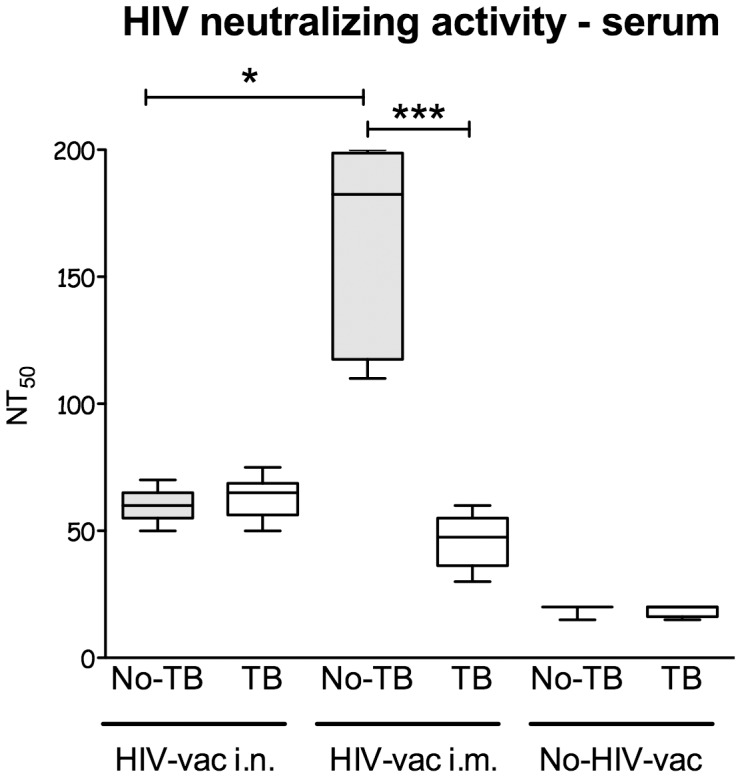
Effect of *Mtb* infection on MultiHIV DNA/protein-induced HIV neutralizing activity in mouse sera. Sera from uninfected and *Mtb*-infected C57BL/6 mice subsequently vaccinated i.n. or i.m. with MultHIV DNA/protein were collected 4 wk post-vaccination. *Ex vivo* HIV-1 neutralization assay was performed on PHA-stimulated PBMCs using two heterologous HIV-1 strains and serially diluted immune mouse sera as described in Materials and Methods. Neutralization titer is defined as reciprocal dilution of serum resulting in 50% inhibition of viral infectivity estimated on the basis of HIV p24 antigen production in PBMCs (NT_50_). Median NT_50_ of 6–8 mice/group from one individual experiment is shown as a solid line. The box defines the 75th and 25th percentiles and the whiskers define the maximum and minimum values (*: P<0.05; ***: P<0.001). The HIV neutralizing activity in mouse sera was investigated in two separate experiments.

Using the 50% HIV neutralization assay we found, that i.n. vaccination of mice with MultiHIV DNA/protein elicited moderate neutralizing serum antibody responses whereas i.m. vaccination resulted in 3-fold higher HIV neutralizing activity (Figure 3; P<0.05). However, while concurrent *Mtb* infection had no effect on neutralizing activity in sera of i.n. vaccinated mice, it reduced over 3-fold the neutralizing response in mice that received the HIV vaccine through the i.m. route (P<0.001).

In conclusion, *Mtb* infection not only reduces the amount of antibodies induced by the HIV vaccine, but also impairs the quality of the antibody response to vaccination.

### Concurrent *Mtb* Infection Amplifies the Th1 Bias of Immune Reponses elicited by HIV Vaccine

**Figure 4:**
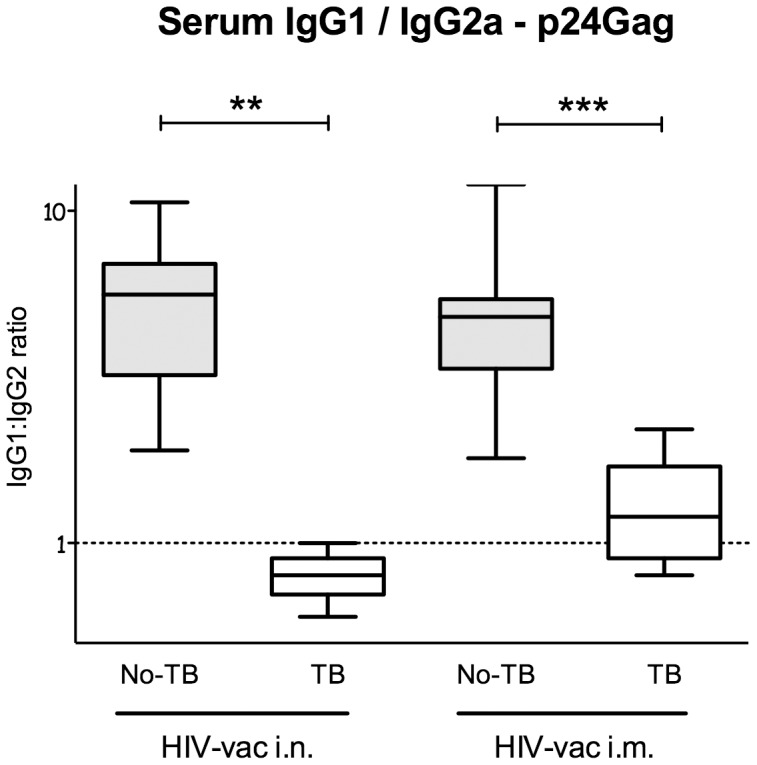
IgG1:IgG2a ratios of HIV p24Gag-specific antibodies in sera of uninfected and *Mtb*-infected mice vaccinated with MultiHIV DNA/protein. Uninfected C57BL/6 mice, or mice infected with *Mtb* via the respiratory route, were immunized and boosted with MultiHIV DNA/protein, as described in Materials and Methods. IgG1:IgG2a ratios were calculated using p24Gag-specific antibody titers determined with ELISA in sera 4 wk post-vaccination. Median IgG1:IgG2a ratio of 6–8 mice/group from one individual experiment is shown as a solid line. The box defines the 75th and 25th percentiles and the whiskers define the maximum and minimum values (**: P<0.01; ***: P<0.001). The IgG1:IgG2a ratios were determined in two separate experiments.

Assessment of vaccine elicited production of IgG1 versus IgG2a indirectly measures differential Th2-Th1 immune reponses and may provide clues that could explain the reduced virus-neutralizing activity in *Mtb*-infected mice. In order to investigate in more detail the impact of concurrent *Mtb* infection on Th2-Th1responses elicited by the HIV vaccine, p24Gag-specific serum IgG1 and IgG2a were determined 4 wk post-vaccination. Uninfected mice inoculated with the HIV vaccine through either the i.n. or the i.m. route had high p24Gag-specific serum IgG1:IgG2a ratios, indicative of immune response with a prevalent Th2 component (Figure 4). Importantly, *Mtb* infection prior to HIV vaccination resulted in 5-fold reduced IgG1:IgG2a ratios regardless of the vaccination route (P<0.001).

Here we show that the host immune response initiated by *Mtb* infection adversely affects the IgG1 : IgG2a ratio elicited by the HIV vaccine. This may explain the reduced neutralizing activity in sera that we observed in *Mtb*-infected HIV vaccinated animals.

### T Cell Responses to MultiHIV DNA/Protein Are Suppressed in Mice Infected with *Mtb* Prior to Vaccination

**Figure 5:**
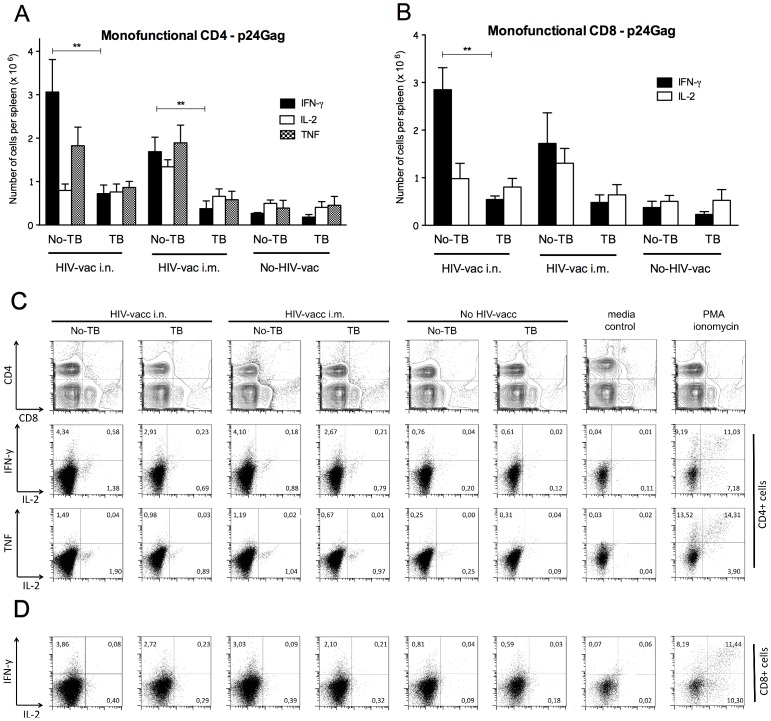
HIV p24Gag-specific T cell cytokine responses in spleens of uninfected or *Mtb*-infected mice vaccinated with MultiHIV DNA/protein. Splenocytes from *Mtb*-infected or uninfected mice, which were immunized and boosted with MultiHIV DNA/protein, were isolated 4 wk post-vaccination, restimulated *ex vivo* with p24Gag antigen, and stained for CD4 and CD8 markers and for intracellular IFN-γ, TNF, and IL-2 (details in Materials and Methods). Numbers of p24Gag-specific CD4+ (A) and CD8+ (B) T cells producing one of three cytokines were determined by flow cytometry. Mean number of single cytokine-producing T cells ± SEM are shown (6–8 animals/group from one individual experiment; **: P<0.01). Flow cytometry plots depict representative intracellular cytokine staining of CD4+ (C) and CD8+ (D) T cells. The cytokine profile of p24Gag-specific T cells was determined in two separate experiments.

Induction of T cells expressing Th1 cytokines: IFN-γ, IL-2, or TNF is necessary for efficient protection against intracellular pathogens such as HIV [25]. HIV-specific multifunctional T cells that simultaneously produce more than one cytokine were recently suggested to play an important role in the control of HIV infection [17]. We studied the magnitude and the quality of T cell responses in splenocytes of MultiHIV DNA/protein-immunized mice 4 wk post-vaccination. We analyzed the numbers and frequencies of CD4 and CD8 T cells that expressed intracellular IFN-γ, IL-2, or TNF, upon restimulation with HIV p24Gag peptide pools *ex vivo*. We found that i.n. vaccination of uninfected mice induced high numbers of p24Gag-specific CD4 (Figure 5A and C) and CD8 (Figure 5B and D) T cells expressing IFN-γ or TNF and low to moderate numbers of IL-2 producing cells as compared to the non-vaccinated controls. The numbers of p24Gag-specific single cytokine producing T cells in i.m. vaccinated mice did not significantly differ from those in mice vaccinated by the i.n. route.

In contrast to non-*Mtb* infected HIV-vaccinated mice, *Mtb*-infected mice had significantly fewer cytokine producing splenic T cells (Figure 5A and B), regardless of the vaccination route. In *Mtb*-infected animals vaccinated i.n., p24Gag-specific IFN-γ-producing CD4 T cells were reduced 4-fold (P<0.01) and TNF-producing CD4 T cells were reduced 2-fold compared to uninfected controls. Similarly, in *Mtb*-infected animals vaccinated i.m., p24Gag-specific IFN-γ-producing CD4 T cells were reduced 4.5-fold (P<0.01) and TNF-producing CD4 T cells were reduced 3-fold compared to uninfected control mice (Figure 5A). A similar decrease of IFN-γ-producing T cells in *Mtb*-infected mice was found in the CD8 T cell subset (5-fold for i.n. route; P<0.01, and 3.5-fold for the i.m. route; Figure 5B). Even though the difference did not reach statistical significance, *Mtb*-infection seemed to negatively influence IL-2 production by antigen-specific CD4 and CD8 T cells following i.m., but not i.n., vaccination (Figure 5).

**Figure 6:**
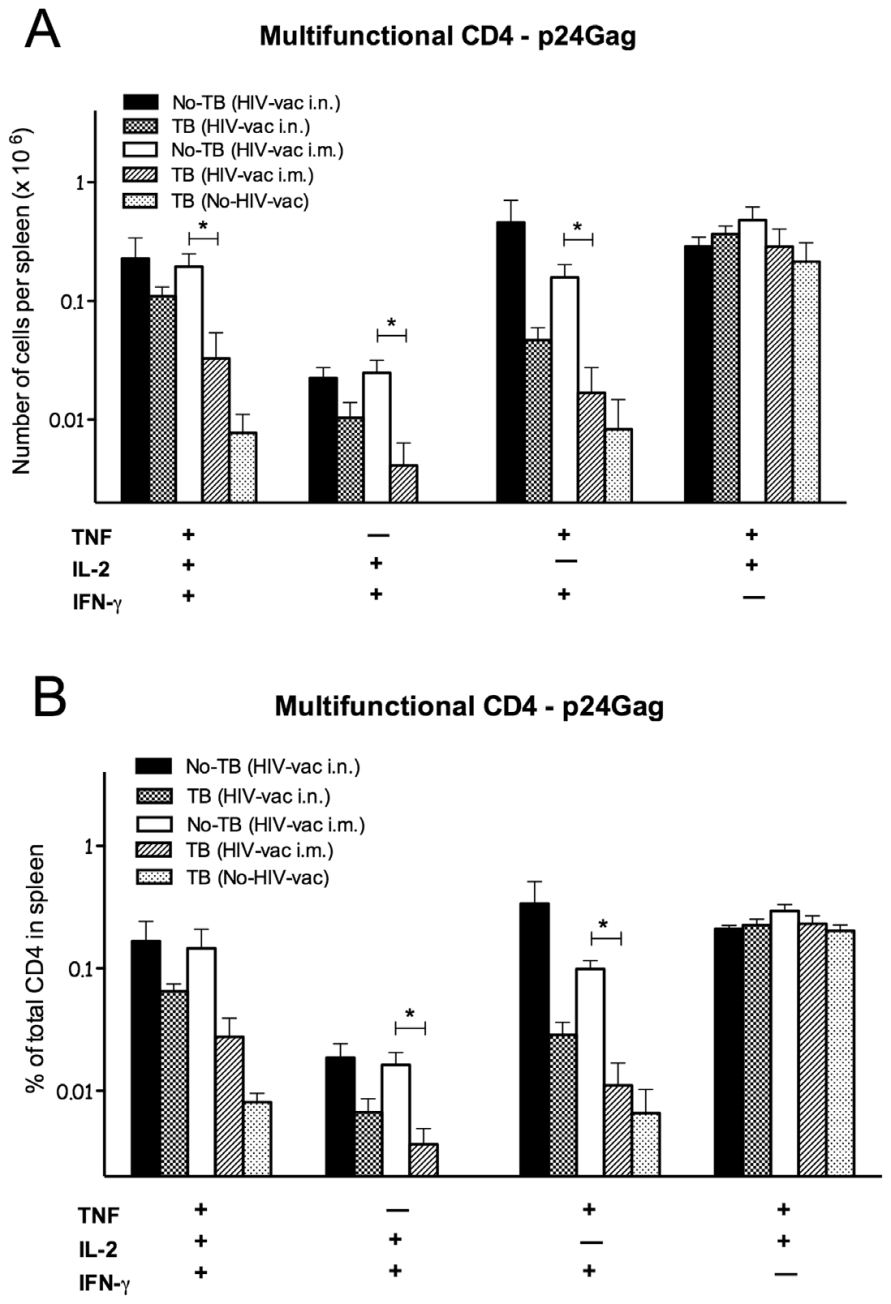
Multifunctional HIV p24 antigen-specific T cells in spleens of uninfected or *Mtb*-infected mice vaccinated with MultiHIV DNA/protein. Splenocytes from *Mtb*-infected or uninfected mice, subsequently immunized and boosted with MultiHIV DNA/protein, were isolated 4 wk post-vaccination, restimulated *ex vivo* with HIV p24 antigen, and stained for CD4 markers and for intracellular IFN-γ, TNF, and IL-2 (details in Materials and Methods). Frequencies of HIV p24 antigen-specific multifunctional CD4 T cells simultaneously producing IFN-γ, IL-2 and TNF were determined by flow cytometry. Mean numbers (A) and frequencies (B) ± SEM of multifunctional T cells are shown (6–8 animals/group from one individual experiment; *: P<0.05). The presence of multifunctional T cells was examined in two separate experiments.

In addition to CD4 and CD8 T cells producing single cytokines, spleens of uninfected HIV-vaccinated mice contained measurable levels of p24Gag-specific multifunctional CD4 T cells which simultaneously expressed IFN-γ/IL-2, IFN-γ/TNF, IL-2/TNF, or IFN-γ/TNF/IL-2 and were similar in mice vaccinated through the i.n. and the i.m. route (Figure 6A). Importantly, both numbers (Figure 6A) and proportions (Figure 6B) of CD4 multifunctional T cells were significantly decreased in spleens of mice that harbored *Mtb* infection at the time of HIV vaccination through the i.m. route, compared to uninfected HIV-vaccinated animals; 6-fold reduction of IFN-γ/IL-2/TNF, and 9-fold reduction of IFN-γ/TNF expressing CD4 T cells were found. A similar trend of diminished levels of IFN-γ/IL-2/TNF and IFN-γ/TNF cells was noted in spleens of i.n. vaccinated mice that were *Mtb*-infected prior to HIV-vaccination. In contrast, *Mtb* infection prior to HIV vaccination did not affect levels of IL-2/TNF expressing p24Gag-specific CD4 T cells (Figure 6). Similarly to the virus-specific humoral response induced by the HIV vaccine, *Mtb* infection has an adverse effect on HIV-specific T cell immunity.

In conclusion, our data show that *Mtb* negatively influences multiple effector functions believed to be important for efficient control of HIV infection.

### Mice Aerosol-Infected with a Low *Mtb* Dose Develop Chronic TB Infection Unaffected by Subsequent HIV Vaccination

In order to evaluate if HIV vaccine may interfere with the course of *Mtb* infection we infected resistant C57BL/6 mice with a low dose of aerosolized *Mtb* via the respiratory route (50–100 bacteria/lung) followed by vaccination with MultiHIV DNA by the i.n. or the i.m. route. Throughout the entire experimental period *Mtb*-infected mice remained in good physical condition and did not show any symptoms of disease. As expected, at the end of the experiment (17 wk post-infection) lungs of all infected mice contained between 4.8–6.0 logs bacteria (Figure S2). Lung bacterial burdens of mice that post-infection received MultiHIV DNA/protein vaccine did not significantly differ from those of non-vaccinated mice irrespective of vaccination route.

## Discussion

It has become increasingly clear that for an effective HIV-1 vaccine to materialize two issues will be critical: a durable antibody response with broad-neutralizing capacity to block the virus transmission [22] and a robust cellular response to limit virus replication in those who already are infected [26]. Using two vaccination routes we investigated the impact of concurrent *Mtb* infection on the immunogenicity of a HIV DNA/protein vaccine candidate in *Mtb* resistant C57BL/6 mice. Remarkably, we found that this subclinical chronic *Mtb* infection impaired both the magnitude and the quality of antibody and T cell responses to the vaccine components p24Gag and gp160Env. Thus, *Mtb*-infected mice showed significant albeit transient decrease of p24Gag-specific serum IgG titers elicited by i.n. vaccination and sustained reduction of both p24Gag- and gp160Env-specific serum IgA induced by i.m. vaccination. Importantly, although concurrent *Mtb* infection in i.m. vaccinated mice did not affect HIV-specific serum IgG levels, it resulted in a significant decrease in serum neutralizing activity towards heterologous HIV clades. Also, *Mtb*-infected mice had, irrespective of the HIV vaccination route, fewer p24Gag-specific IFN-γ- or TNF-producing splenic T cells, and, importantly, reduced levels of p24Gag-specific multifunctional T cells simultaneously expressing IFN-γ, TNF, and IL-2.

Thus, in our study, pre-existing subclinical chronic *Mtb* infection in mice interfered with many types of immune responses to HIV antigens which are considered important for both natural and vaccine-induced immunity, such as mucosal and serum antibodies, including neutralizing antibodies [27], [16], robust CD4 and CD8 T cell responses [17], [20], and multifunctional T cells [28]. This is, to our knowledge, the first report on detrimental effect of *Mtb* infection on development of post-vaccination HIV immunity. Furthermore, this is also the first report on impairment by mycobacteria of immune responses to any type of heterologous antigens, with the exception of an early study by Dubos et al who showed, that infection of mice with BCG or administration of mycobacterial lipids could, depending on the administration route, either protect from or enhance concurrent infection with *Staphylococcus aureus* [29], [30].

It has previously been reported that some chronic and acute infections other than TB, including those with intracellular pathogens, may modify immune responses to and efficacy of heterologous vaccines. Helminth infections have been implicated in a compromised protective efficacy of tetanus [31] and BCG vaccinations [32]. Malaria has been suggested to suppress antibody responses to meningococcal C [33] and *Salmonella typhi* O [34] polysaccharide vaccines and tetanus vaccine [35]. Immunosuppressive effects of malaria have also been associated with increased risk of bacterial infections [36] and increased HIV loads [37]. Similarly, certain viral infections, have been found to interfere with immune responses to unrelated vaccines and concurrent infections; attenuated polio virus administered with oral polio vaccine has been associated with reduced efficacy of BCG vaccination [38], measles was found to non-specifically suppress immune responses to secondary bacterial infections for instance *Listeria monocytogenes* [39] and HIV infection has been shown to increase the risk of mycobacterial diseases including TB [12].

The mechanisms underlying the ability of certain pathogens to impair immune responses to heterologous vaccines or concurrent infections, including detrimental effect of *Mtb* infection on HIV vaccination described here, remain as yet unclear. In this study we found that chronic Mtb infection impairs both humoral and cellular immune responses elicited by HIV vaccination; therefore we expect that more than one mechanism is involved. One of the better-explored mechanisms of pathogen-related immune subversion is exerted by helmithes and protozoa and relies on an induction of a highly Th2 cell-polarized environment. Because of the crossregulation between Th1 and Th2 responses, the helminth-induced pertinent Th2 bias may result in the increased susceptibility to Th1-controlled infections [40], [41] and decreased responses to vaccinations [31], [32], [42]. However, this mechanism does not account for the *Mtb*-mediated suppression of HIV vaccine-induced responses reported here because, instead of increased Th2 polarization, we observed augmented Th1 bias in mice infected with *Mtb* prior to HIV vaccination.

Studies of HIV positive subjects reveal the presence of both neutralizing antibodies and serum antibodies that will enhance HIV infection, the latter with opsonizing activity allowing the virus to enter and multiply inside host cells [43] The balance between two types of antibodies changes as the disease progresses and during advanced stages the proportion of infection-enhancing opsonizing antibodies are generally higher than neutralizing anibodies. It could be hypothesized that *Mtb* infection drives mainly production of opsonizing IgG antibodies. This could be the reason why despite high serum levels of IgG elicited by i.m. HIV vaccination in both *Mtb*-infected and non-infected mice, serum antibodies of infected animals had much lower neutralizing ability than in non *Mtb*-infected counterparts.

Since *Mtb* and HIV do not share antigenic epitopes, it is unlikely that the observed inhibition by concurrent TB of immune responses to the HIV vaccine candidate is antigen-specific. Instead, it could be envisaged that non-antigen specific “bystander” suppressive mechanisms are involved in such inhibition. Indeed, evidence supports the idea that infection-induced specific regulatory T cells, in addition to suppression of specific immune responses, can also suppress unrelated immune responses in a non-antigen specific manner, either through direct cellular contact or via the regulatory cytokines they produce [44]–[46]. Regulatory T cells have been shown to be induced both in active and latent TB [47] and have been implicated in the downregulation of immune control of *Mtb* infection and progression to active disease [48], [49]. Additionally, the impairment of humoral and T cell responses to intranasal HIV vaccination which we found in *Mtb*-infected mice could also result from a competitive presentation of *Mtb* antigens and HIV vaccine antigens co-localizing in respiratory tract-associated lymph nodes [50].

In summary, we report for the first time that chronic *Mtb* infection of mice prior to inoculation with an experimental HIV vaccine has detrimental effect on vaccine-specific antibody and T cell responses. These results suggest that asymptomatic *Mtb* infection could also interfere with prospective HIV vaccination in humans. Therefore, we firmly believe that our findings have important implications for the development of potential HIV candidates. When ultimately a HIV vaccine is available, the need for such a vaccine will be greatest where TB is endemic. As estimated by the WHO currently one third of the world’s population is latently infected with *Mtb* [51]. This vast number of latently infected constitutes the main reservoir for adult pulmonary TB; in about 5–10% of such individuals the infection becomes reactivated mainly when the immune system is compromised [52]. Recent studies on mutation rates of *Mtb* bacilli suggest that during the latent stage of infection, the bacteria are able to slowly replicate [53]–[54]. This may lead to low level engagement of the immune system [55]. Studies of low dose *Mtb* aerosol infections in macaques, resulting in approximately half of the monkeys being classified as latently infected that may naturally reactivate, and observations from epidemiological studies strongly suggest that the transition from latent to active TB is a multistage and gradual process [56]–[58]. Together with other factors this extended subclinical phase could result in a delayed or false negative diagnosis [59]. Therefore, our results advocate that chronic and undiagnosed, or even latent, *Mtb* infection interferes with the immune response elicited by HIV vaccine candidates and should be taken into account during vaccine design and in clinical trials.

## Materials and Methods

### *Mtb* Infection

*Mtb* Harlingen strain was prepared as described earlier [60]. Six wk-old female C57BL/6 mice were infected aerogenically with low dose of *Mtb* (50–100 bacteria/lung) using a nose-only aerosol exposure apparatus (In-Tox Products, Moriarty, NM, USA) as previously described [61].

Lung inoculum was verified by agar plating 24 h after infection. Lungs for all time points were homogenized in PBS with 0.02% Tween 80 and serial dilutions of lung homogenates were plated onto Middlebrook 7H10 agar. Colonies were counted after 2 to 3 wk incubation at 37°C. All work with *Mtb* and *Mtb*-infected animals was conducted in a BSL-3 containment laboratory. The local committee on animal ethics and the Swedish Board of Agriculture approved all animal experiments.

### HIV Vaccine and Immunization

Seven wk post-*Mtb* infection groups of mice (6–8 animals/group) were immunized i.n. or i.m. with 10 µg of plasmids encoding HIV-1 subtype B gp160Env, p37Gag, Nef, Tat, and Rev (MultiHIV DNA vaccine) [24] formulated in N3 adjuvant (2% lipid) and subsequently boosted twice (4 and 6 wk post-DNA vaccination) with 5 µg of recombinant gp160Env, p24Gag, Tat and Nef formulated in L3 adjuvant (2% lipid) [62], [63]. Control groups were sham-vaccinated with saline.

### Sample Collection

Two wk after the last HIV boost vaccination tail blood and vaginal wash samples were collected for IgG and IgA assays and frozen until the time of analysis. Four wk post-vaccination the mice were sacrificed and blood, vaginal washes, spleens and lungs were collected.

### In Vitro Stimulation and Intracellular Cytokine Staining

A single cell suspension of splenocytes was obtained by grinding the spleen and passing the obtained cell suspension through a 70 µm strainer (BD Falcon) into DMEM (BD Bioscience) supplemented with 10% inactivated FBS, penicillin/streptomycin, L-glutamine, sodium-pyruvate (Invitrogen). Erythrocytes were lysed with NH_4_Cl and the remaining splenocytes (1×10^6^/well) from individual animals were stimulated for 6 h in the presence of peptide pools (15-mers overlapping by 10 amino acids, Thermo-Hybaid, Germany, 1.25 µg/ml each peptide) covering either gp160Env or p24Gag proteins dissolved in complete DMEM medium, in the presence of brefeldin A (10 µg/mL, eBioscience). Medium alone was used as a negative control and PMA/Ionomycin (at 25 ng/mL and 1 µg/mL, Sigma-Aldrich) was used as positive control. After stimulation the cells were washed with FACS buffer (PBS with 1% FBS) and incubated with purified anti-mouse CD16/CD32 (2.4G2, BD Bioscience) at 20 µg/mL for 15 min at 4°C to block nonspecific binding (Fc block). The cells were washed and incubated 15 min at 4°C with primary antibodies specific for surface makers (anti-CD3 17A2, anti-CD4 GK1.5, and anti-CD8α 53-6.7, all from eBioscience), or appropriate isotype controls, diluted in FACS buffer. After washing, cells were fixed with 2% paraformaldehyde, permeabilized using permeabilization buffer (eBioscience) and incubated for 20 min at 20°C with antibodies specific for intracellular cytokines; anti-IL-2 JE56-5H4, anti-IFN-γ XMG1.2, and anti-TNF-α MP6-XT22, all from eBioscience. The cells were washed with permeabilization buffer and then with FACS buffer, resuspended and analyzed by flow cytometry using BD FACSCanto II flow cytometer (BD Biosciences). Data analysis was performed using FlowJo software.

### IgG and IgA ELISA

Vaginal washes were obtained and analyzed as previously described [64]–[66]. 96-well plates (Nunc Maxisorp) were coated with the recombinant HIV-1 proteins p24Gag (0.5 µg/mL, Aalto Bio Reagents), gp160Env (0.5 µg/mL, BioSciences Int), Nef (0.5 µg/mL, kindly provided by V. Erfle, GSF, Munich, Germany), and Tat (1 µg/mL, kindly provided by C. Svanholm, KI, Stockholm). ELISA was carried out essentially as previously described [67].

### Neutralization Assay

Immune mouse sera were pooled within each experimental group and were tested for the presence of neutralizing activity. Sera were heat-inactivated (56°C for 30 min) and serially diluted at 3-fold dilutions, starting at 1/20. Neutralization assay was performed as described earlier using replication of HIV-1 SF2 strain and the primary NSI/CCR5 tropic clade B isolate 6920 in PBMCs as readout system [65]. Virus production was measured in a p24Gag antigen capture ELISA [25]. An HIV-1-positive serum pool (HIVIG) and the human mAb 2F5, specific for the gp41 ELDKWAS epitope, were used as a positive control. Neutralization was defined as the sample titer resulting in 50% reduction (NT_50_) of p24Gag antigen in the supernatant compared with p24Env antigen content when the virus was incubated in the presence of HIV Ab from negative serum. All assays were repeated at least twice.

### Statistical Analysis

The statistical significance of differences between groups was calculated by the one-way nonparametric Kruskal-Wallis test followed by Dunn’s multiple comparison posttest IgG1/IgG2a ratios were compared using a two-tailed Student *t* test. Statistical analysis was performed using GraphPad PRISM software version 5.0 (GraphPad Software, Inc). P-values were considered to be significant if less than 0.05. Experiments were repeated twice.

## Supporting Information

Figure S1 **Effect of** ***Mtb*** **infection on HIV-specific serum IgG titers induced in mice by MultiHIV DNA vaccination followed by protein boost.** Uninfected or *Mtb*-infected C57BL/6 mice were immunized and boosted with MultiHIV DNA/protein, as described in Materials and Methods. 4 wk post-vaccination HIV-specific serum IgG levels were assayed with HIV antigen-ELISA using Tat and Nef as coating antigens, as described in Materials and Methods. Median endpoint titer of 6–8 mice/group from one individual experiment is shown as a solid line. The box defines the 75th and 25th percentiles and the whiskers define the maximum and minimum values. Dashed line indicates the ELISA sensitivity threshold. HIV-specific serum IgG levels were assayed in two separate experiments.
(TIF)

Figure S2 **Bacterial loads in lungs of mice 17 wk post-infection with** ***Mtb*** **.** C57BL/6 mice aerogenically infected with low dose *Mtb* (50–100 bacteria/lung) were, 7 wk later, vaccinated i.n. or i.m. with MultiHIV DNA in N3 adjuvant followed by two booster inoculations of HIV proteins in L3 adjuvant (details in Materials and Methods). Control group of mice was left unvaccinated. Lung homogenates from mice sacrificed 17 wk post-infection were plated on Middlebrook agar and bacterial CFU were enumerated as described in Materials and Methods. Median lung CFU value of 6–8 mice/group from individual experiment is shown as a solid line. The box defines the 75th and 25th percentiles and the whiskers define the maximum and minimum values. The bacterial load was determined in two separate experiments.
(TIF)

## References

[1] AIDS epidemic update November 2009, UNAIDS, WHO. http://data.unaids.org:80/pub/Report/2009/JC1700_Epi_Update_2009_en.pdf.

[2] Talbot HK, Libster R, Edwards K (2012). Influenza vaccination for older adults.. Hum Vaccin Immunother 8: [Epub ahead of print].

[3] Iyer SS, Chatraw JH, Tan WG, Wherry EJ, Becker TC (2012). Protein energy malnutrition impairs homeostatic proliferation of memory CD8 T cells.. J Immunol.

[4] Whittle HC, Bradley-Moore A, Fleming A, Greenwood BM (1973). Effects of measles on the immune response of Nigerian children.. Arch Dis Child.

[5] Williamson WA, Greenwood BM (1978). Impairment of the immune response to vaccination after acute malaria.. Lancet.

[6] Cunnington AJ, Riley EM (2010). Suppression of vaccine responses by malaria: insignificant or overlooked?. Expert Rev Vaccines.

[7] Usen S, Milligan P, Ethevenaux C, Greenwood B, Mulholland K (2000). Effect of fever on the serum antibody response of Gambian children to *Haemophilus influenzae* type b conjugate vaccine.. Pediatr Infect Dis J.

[8] Dauby N, Alonso-Vega C, Suarez E, Flores A, Hermann E (2009). Maternal infection with *Trypanosoma cruzi* and congenital Chagas disease induce a trend to a type 1 polarization of infant immune responses to vaccines.. PloS Negl Trop Dis.

[9] Urban Jr JF, Steenhard NR, Solano-Aguilar GI, Dawson HD, Iweala OI (2007). Infection with parasitic nematodes confounds vaccination efficacy.. Vet Parasitol.

[10] Harries AD, Zachariah R, Corbett EL, Lawn SD, Santos-Filho ET (2010). The HIV-associated tuberculosis epidemic-when will we act?. Lancet.

[11] Pawlowski A, Jansson M, Sköld M, Rottenberg ME, Källenius G (2012). Tuberculosis and HIV co-infection.. PLoS Pathog.

[12] Diedrich CR, Flynn JL (2011). HIV/*M. tuberculosis* co-infection immunology: How does HIV exacerbate TB?. Infect Immun.

[13] Lawn SD, Butera ST, Folks TM (2001). Contribution of immune activation to the pathogenesis and transmission of human immunodeficiency virus type 1 infection.. Clin Microbiol Rev.

[14] Cingolani A, Cozzi Lepri A, Castagna A, Goletti D, De Luca A (2012). Impaired CD4 T-cell count response to combined antiretroviral therapy in antiretroviral-naive HIV-infected patients presenting with tuberculosis as AIDS-defining condition.. Clin Infect Dis.

[15] Poropatich K, Sullivan DJ (2011). Human immunodeficiency virus type 1 long-term non-progressors: the viral, genetic and immunological basis for disease non-progression. J Gen Virol..

[16] Masopust D (2009). Developing an HIV cytotoxic T-lymphocyte vaccine: issues of CD8 T-cell quantity, quality and location.. J Intern Med.

[17] Owen RE, Heitman JW, Hirschkorn DF, Lanteri MC, Biswas HH (2010). HIV+ elite controllers have low HIV-specific T-cell activation yet maintain strong, multifunctional T-cell responses.. AIDS.

[18] Tomaras GD, Haynes BF (2010). Strategies for eliciting HIV-1 inhibitory antibodies.. Curr Opin HIV AIDS.

[19] Mikell I, Sather DN, Kalams SA, Altfeld M, Alter G (2011). Characteristics of the earliest cross-neutralizing antibody response to HIV-1. PLoS Pathog..

[20] Belyakov IM, Ahlers JD (2012). Mucosal immunity and HIV-1 infection: applications for mucosal AIDS vaccine development.. Curr Top Microbiol Immunol.

[21] Perrin H, Canderan G, Sékaly RP, Trautmann L (2010). New approaches to design HIV-1 T-cell vaccines.. Curr Opin HIV AIDS.

[22] Kim JH, Rerks-Ngarm S, Excler JL, Michael NL (2010). HIV vaccines: lessons learned and the way forward.. Curr Opin HIV AIDS.

[23] Rerks-Ngarm S, Pitisuttithum P, Nitayaphan S, Kaewkungwal J, Chiu J (2009). Vaccination with ALVAC and AIDSVAX to prevent HIV-1 infection in Thailand.. N Engl J Med.

[24] Sandström E, Nilsson C, Hejdeman B, Bråve A, Bratt G, et al (2008). Broad immunogenicity of a multigene, multiclade HIV-1 DNA vaccine boosted with heterologous HIV-1 recombinant modified vaccinia virus Ankara.. J Infect Dis.

[25] Foulds KE, Wu CY, Seder RA (2006). Th1 memory: implications for vaccine development.. Immunol Rev.

[26] Haynes BF, Liao HX, Tomaras GD. Is developing an HIV-1 vaccine possible? (2010). Curr Opin HIV AIDS.

[27] Gizurarson S, Sigurdoardóttir M, Stanzeit B (1998). Selective augmentation of antibodies in various mucosal regions, after intranasal immunization with diphtheria in mice.. J Pharm Sci.

[28] Burgers WA, Chege GK, Müller TL, van Harmelen JH, Khoury G (2009). Broad, high-magnitude and multifunctional CD4+ and CD8+ T-cell responses elicited by a DNA and modified vaccinia Ankara vaccine containing human immunodeficiency virus type 1 subtype C genes in baboons.. J Gen Virol.

[29] Dubos RJ, Schaedler RW (1957). Effects of cellular constituents of mycobacteria on the resistance of mice to heterologous infections I. Protective effects.. J Exp Med.

[30] Schaedler RW, Dubos RJ (1957). Effects of cellular constituents of mycobacteria on the resistance of mice to heterologous infections. II. Enhancement of infection.. J Exp Med.

[31] Nookala S, Srinivasan S, Kaliraj P, Narayanan RB, Nutman TB (2004). Impairment of tetanus-specific cellular and humoral responses following tetanus vaccination in human lymphatic filariasis.. Infect Immun.

[32] Elias D, Akuffo H, Pawlowski A, Haile M, Schön T (2005). *Schistosoma mansoni* infection reduces the protective efficacy of BCG vaccination against virulent *Mycobacterium tuberculosis*.. Vaccine.

[33] Greenwood BM, Bradley AK, Blakebrough IS, Whittle HC, Marshall TF (1980). The immune response to a meningococcal polysaccharide vaccine in an African village.. Trans R Soc Trop Med Hyg.

[34] Bradley-Moore AM, Greenwood BM, Bradley AK, Bartlett A, Bidwell DE (1985). Malaria chemoprophylaxis with chloroquine in young Nigerian children. II. Effect on the immune response to vaccination.. Ann Trop Med Parasitol.

[35] McGregor IA, Barr M (1962). Antibody response to tetanus toxoid inoculation in malarious and non-malarious Gambian children.. Trans R Soc Trop Med Hyg.

[36] Bassat Q, Guinovart C, Sigauque B, Mandomando I, Aide P (2009). Severe malaria and concomitant bacteraemia in children admitted to a rural Mozambican hospital.. Trop Med Int Health.

[37] Kublin JG, Patnaik P, Jere CS, Miller WC, Hoffman IF (2005). Effect of *Plasmodium falciparum* malaria on concentration of HIV-1-RNA in the blood of adults in rural Malawi: a prospective cohort study.. Lancet.

[38] Sartono E, Lisse IM, Terveer EM, van de Sande PJ, Whittle H (2010). Oral polio vaccine influences the immune response to BCG vaccination. A natural experiment.. PLoS One.

[39] Slifka MK, Homann D, Tishon A, Pagarigan R, Oldstone MB (2003). Measles virus infection results in suppression of both innate and adaptive immune responses to secondary bacterial infection.. J Clin Invest.

[40] Elias D, Akuffo H, Thors C, Pawlowski A, Britton S (2005). Low dose chronic *Schistosoma mansoni* infection increases susceptibility to *Mycobacterium bovis* BCG infection in mice.. Clin Exp Immunol.

[41] Hartgers FC, Yazdanbakhsh M (2006). Co-infection of helminths and malaria: modulation of the immune responses to malaria.. Parasite Immunol.

[42] Cooper PJ, Chico M, Sandoval C, Espinel I, Guevara A (2001). Human infection with *Ascaris lumbricoides* is associated with suppression of the interleukin-2 response to recombinant cholera toxin B subunit following vaccination with the live oral cholera vaccine CVD 103-HgR.. Infect Immun.

[43] Subbramanian RA, Xu J, Toma E, Morisset R, Cohen EA (2002). Comparison of human immunodeficiency virus (HIV)-specific infection-enhancing and -inhibiting antibodies in AIDS patients.. J Clin Microbiol.

[44] Thornton AM, Shevach EM (2000). Suppressor effector function of CD4+CD25+ immunoregulatory T cells is antigen nonspecific.. J Immunol.

[45] von Boehmer H (2005). Mechanisms of suppression by suppressor T cells.. Nat Immunol.

[46] Hu G, Liu Z, Zheng C, Zheng SG (2010). Antigen-non-specific regulation centered on CD25+Foxp3+ Treg cells.. Cell Mol Immunol.

[47] Marin ND, París SC, Vélez VM, Rojas CA, Rojas M (2010). Regulatory T cell frequency and modulation of IFN-gamma and IL-17 in active and latent tuberculosis.. Tuberculosis (Edinb).

[48] Kursar M, Koch M, Mittrücker HW, Nouailles G, Bonhagen K (2007). Cutting Edge: Regulatory T cells prevent efficient clearance of *Mycobacterium tuberculosis*.. J Immunol.

[49] Chen X, Zhou B, Li M, Deng Q, Wu X (2007). CD4(+)CD25(+)FoxP3(+) regulatory T cells suppress *Mycobacterium tuberculosis* immunity in patients with active disease.. Clin Immunol.

[50] Balmelli C, Demotz S, Acha-Orbea H, De Grandi P, Nardelli-Haefliger D (2002). Trachea, lung, and tracheobronchial lymph nodes are the major sites where antigen-presenting cells are detected after nasal vaccination of mice with human papillomavirus type 16 virus-like particles.. J Virol.

[51] World Health Organization (2009). Global tuberculosis control: a short update to the 2009 report. Geneva: World Health Organization.. vi, 39 p.

[52] Lin PL, Flynn JL (2010). Understanding latent tuberculosis: a moving target.. J Immunol.

[53] Sherman DR, Gagneux S (2011). Estimating the mutation rate of *Mycobacterium tuberculosis* during infection.. Nat Genet.

[54] Ford CB, Lin PL, Chase MR, Shah RR, Iartchouk O (2011). Use of whole genome sequencing to estimate the mutation rate of *Mycobacterium tuberculosis* during latent infection.. Nat Genet.

[55] Cardona PJ (2009). A dynamic reinfection hypothesis of latent tuberculosis infection.. Infection.

[56] Capuano SV, Croix DA, Pawar S, Zinovik A, Myers A (2003). Experimental *Mycobacterium tuberculosis* infection of cynomolgus macaques closely resembles the various manifestations of human *M. tuberculosis* infection.. Infect Immun.

[57] Lin PL, Rodgers M, Smith L, Bigbee M, Myers A (2009). Quantitative comparison of active and latent tuberculosis in the cynomolgus macaque model.. Infect Immun.

[58] Mtei L, Matee M, Herfort O, Bakari M, Horsburgh, C R, Waddell R, Cole, B F (2005). High rates of clinical and subclinical tuberculosis among HIV-infected ambulatory subjects in Tanzania.. Clin Infect Dis.

[59] Cunningham J, Perkins M (2006). Diagnostics for tuberculosis: global demand and market potential. WHO.. Geneva, Switzerland.

[60] Hamasur B, Haile M, Pawlowski A, Schröder U, Williams A (2003). *Mycobacterium tuberculosis* arabinomannan-protein conjugates protect against tuberculosis.. Vaccine.

[61] Leepiyasakulchai C, Ignatowicz L, Pawlowski A, Källenius G, Sköld M (2012). Failure to recruit anti-inflammatory CD103+ dendritic cells and a diminished CD4+ Foxp3+ regulatory T cell pool in mice that display excessive lung inflammation and increased susceptibility to *Mycobacterium tuberculosis*.. Infect Immun.

[62] Hinkula J, Devito C, Zuber B, Benthin R, Ferreira D (2006). A novel DNA adjuvant, N3, enhances mucosal and systemic immune responses induced by HIV-1 DNA and peptide immunizations.. Vaccine.

[63] Bråve A, Johansen K, Palma P, Benthin R, Hinkula J (2008). Maternal immune status influences HIV-specific immune responses in pups after DNA prime protein boost using mucosal adjuvant.. Vaccine.

[64] Asakura Y, Lundholm P, Kjerrstrom A, Benthin R, Lucht E (1999). DNA-plasmids of HIV-1 induce systemic and mucosal immune responses.. Biol Chem.

[65] Lundholm P, Asakura Y, Hinkula J, Lucht E, Wahren B (1999). Induction of mucosal IgA by a novel jet delivery technique for HIV-1 DNA.. Vaccine.

[66] Devito C, Levi M, Broliden K, Hinkula J (2000). Mapping of B-cell epitopes in rabbits immunised with various gag antigens for the production of HIV-1 gag capture ELISA reagents.. J Immunol Methods.

[67] Devito C, Zuber B, Schröder U, Benthin R, Okuda K (2004). Intranasal HIV-1-gp160-DNA/gp41 peptide prime-boost immunization regimen in mice results in long-term HIV-1 neutralizing humoral mucosal and systemic immunity.. J Immunol.

