# Supplementary figures and images for: Mycobacterium tuberculosis Infection Interferes with HIV Vaccination in Mice
Source: PLoS One. 2012 Jul 23;7(7):e41205. doi: 10.1371/journal.pone.0041205 (PMC3406616; doi:10.1371/journal.pone.0041205)

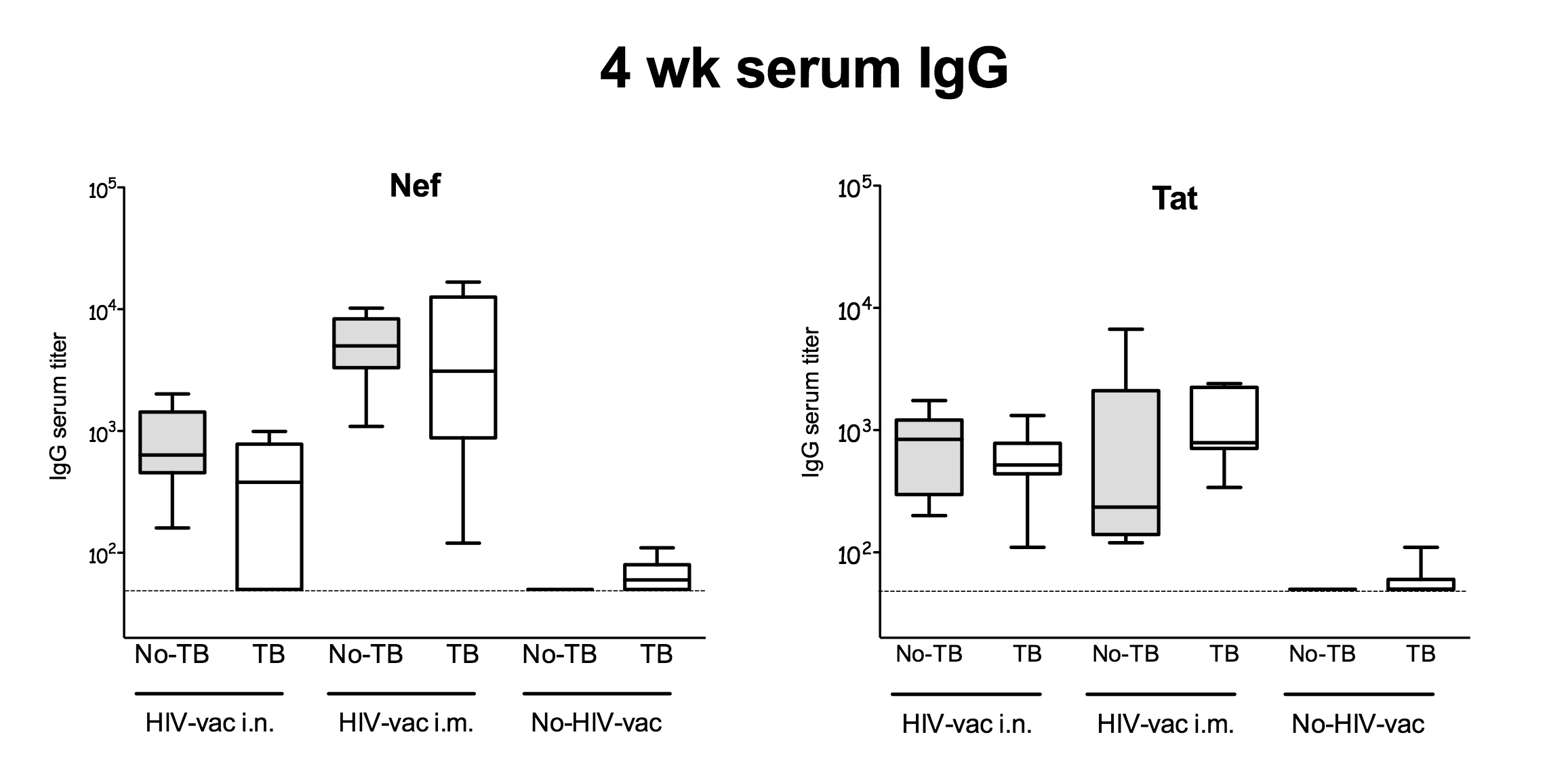

Supplement: Figure S1 — Effect of Mtb infection on HIV-specific serum IgG titers induced in mice by MultiHIV DNA vaccination followed by protein boost. Uninfected or Mtb-infected C57BL/6 mice were immunized and boosted with MultiHIV DNA/protein, as described in Materials and Methods. 4 wk post-vaccination HIV-specific serum IgG levels were assayed with HIV antigen-ELISA using Tat and Nef as coating antigens, as described in Materials and Methods. Median endpoint titer of 6–8 mice/group from one individual experiment is shown as a solid line. The box defines the 75th and 25th percentiles and the whiskers define the maximum and minimum values. Dashed line indicates the ELISA sensitivity threshold. HIV-specific serum IgG levels were assayed in two separate experiments. (TIF) [file pone.0041205.s001.tif]

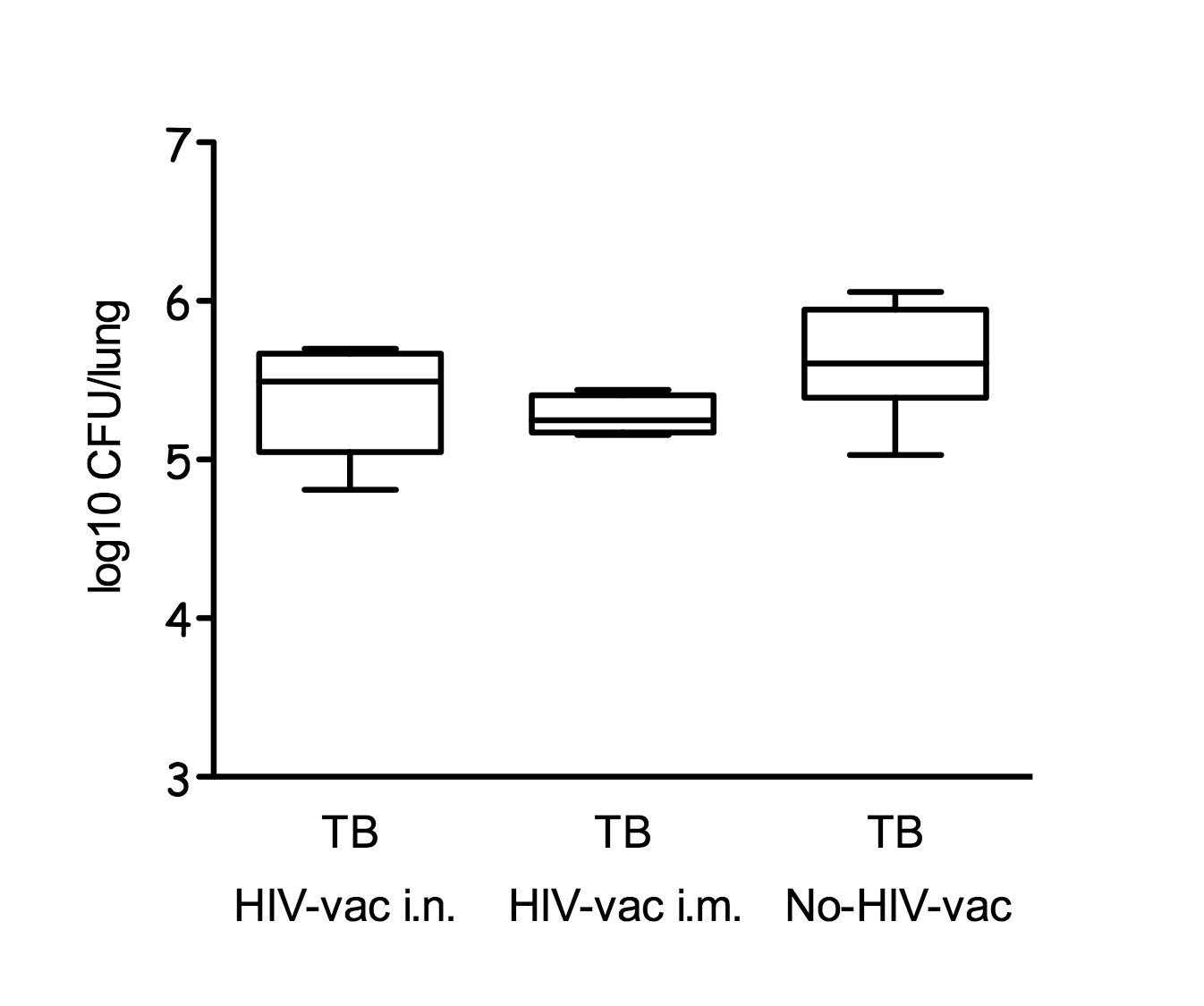

Supplement: Figure S2 — Bacterial loads in lungs of mice 17 wk post-infection with Mtb . C57BL/6 mice aerogenically infected with low dose Mtb (50–100 bacteria/lung) were, 7 wk later, vaccinated i.n. or i.m. with MultiHIV DNA in N3 adjuvant followed by two booster inoculations of HIV proteins in L3 adjuvant (details in Materials and Methods). Control group of mice was left unvaccinated. Lung homogenates from mice sacrificed 17 wk post-infection were plated on Middlebrook agar and bacterial CFU were enumerated as described in Materials and Methods. Median lung CFU value of 6–8 mice/group from individual experiment is shown as a solid line. The box defines the 75th and 25th percentiles and the whiskers define the maximum and minimum values. The bacterial load was determined in two separate experiments. (TIF) [file pone.0041205.s002.tif]
